# Use of waste canola oil as a low-cost substrate for rhamnolipid production using *Pseudomonas aeruginosa*

**DOI:** 10.1186/s13568-019-0784-7

**Published:** 2019-05-06

**Authors:** Beatriz Pérez-Armendáriz, Carlos Cal-y-Mayor-Luna, Elie Girgis El-Kassis, Luis Daniel Ortega-Martínez

**Affiliations:** Department of Biological Sciences, Universidad Popular Autónoma del Estado de Puebla, 13 Poniente No. 1927, Col Barrio de Santiago, C.P. 72410 Puebla, Mexico

**Keywords:** Factorial design, *Pseudomonas aeruginosa*, Rhamnolipid, Waste canola oil

## Abstract

Rhamnolipids are glycolipid biosurfactants that are primarily produced by *Pseudomonas aeruginosa* that have gained a great deal of interest for their numerous industrial applications and environmentally friendly properties. In this study, we explored the potential of waste canola oil as a low-cost and environmentally friendly substrate for the production of rhamnolipids by *P. aeruginosa*. Four different 2^3^ full factorial designs were used to assess the effect of three independent factors on rhamnolipid production, including carbon source (canola oil and waste canola oil), nitrogen source [(NH_4_)_2_SO_4_ and NaNO_3_] and production time (7 and 14 days). The highest observed yield was 3585.31 ± 66.24 mg/L when *P. aeruginosa* was cultured for 14 days with 3% v/v waste canola oil and 4 g/L of NaNO_3_. The nitrogen source proved to be a crucial factor, as the use of NaNO_3_ rather than (NH_4_)_2_SO_4_ led to a 30-fold increase in production yield. The observed yield when waste canola oil was used was similar to, and even slightly higher than, that obtained using canola oil. Our results showed that waste canola oil has great potential for use as a carbon source for rhamnolipid production by *P. aeruginosa*, thus paving the way for the development of a low-cost, efficient, and environmentally friendly bioprocess for the production of rhamnolipids.

**Electronic supplementary material:**

The online version of this article (10.1186/s13568-019-0784-7) contains supplementary material, which is available to authorized users.

## Introduction

Biosurfactants are secondary metabolites with amphiphilic properties that are produced by a variety of microorganisms (Pacwa-Plociniczak et al. 2011). These surface-active molecules have the same properties as their chemical counterparts with respect to their emulsification, detergency and foaming properties, but they are more environmentally friendly (Moya-Ramírez et al. 2015). There is increasing interest in replacing synthetic surfactants with those of biological origin (Camilios-Neto et al. 2008), the latter of which have a variety of advantages, including their ecological acceptability, low environmental toxicity, biodegradability, effectiveness, stability, and activity at high temperatures, extreme pH values and high salinity (George and Jayachandran 2012). These compounds are biosynthesized when microorganisms, especially bacteria, fungi or yeast, are grown in medium containing hydrophobic compounds, such as oil or hydrocarbons (Thavasi et al. 2011). Nevertheless, biosurfactants can also be produced using alternative substrates, such as carbohydrates, glycerol or agro-industrial wastes (Wang et al. 2007).

Glycolipids are the most common biosurfactants in nature (Shoeb et al. 2013) and are formed of mono, di, tri or tetra saccharides of mannose, glucose, galactose or rhamnose that are attached to long-chain aliphatic acids with an ester or ether linkage (Rahman and Gakpe 2008). Rhamnolipids, the most studied of this class of biosurfactants, are anionic glycolipids that are formed of an l-rhamnose residue and units of β-hydroxyalkanoic acids (Müller et al. 2012). The precursors used for rhamnolipids synthesis include the nucleotide activated saccharide dTDP-l-rhamnose and hydrophobic moieties such as 3-(3-hydroxyalkanoyloxy) alkanoic acid (HAA). The sugar moiety can be synthesized from d-glucose, while the hydrophobic moiety can be synthesized through the fatty acid synthesis pathway, starting with two-carbon units (Chong and Li, 2017). HAA, mono- and di-rhamnolipids are almost exclusively synthesized by *Pseudomonas* sp. In the last decades, rhamnolipids have emerged as a promising class of biosurfactants and biotechnological products owing to their unique properties and industrials applications and may represent a sustainable alternative for traditional surfactants (Dobler et al. 2016).

Rhamnolipids have a high surface activity and emulsification index. In addition, they can be produced at relatively high yields by bacteria; further, this approach requires relatively short incubation times and uses a well understood means of production. Furthermore, because rhamnolipids are virulence factors of *Pseudomonas aeruginosa*, many aspects of their biosynthesis have been investigated to control their production and effects (Abdel-Mawgoud et al. 2011).

The industrial production of rhamnolipids has generated a great deal of interest due to their diverse applications in many fields (Chong and Li 2017). For example, rhamnolipids are used as whiteners and detergents because of their natural surface-active properties (Sekhon-Randhawa and Rahman 2014). Rhamnolipids are also used in bioremediation and oil recovery (Sharma et al. 2018) because of their excellent emulsification properties, which make them highly useful in removing crude oil from contaminated soils. With respect to their pharmaceutical and therapeutic applications, rhamnolipids exhibit low toxicity and have antimicrobial properties against pathogens such as *Staphylococcus aureus* and *Listeria monocytogenes* (Chen et al. 2017). In cosmetics, rhamnolipids have been shown to be effective for several skin treatments applications, such as for wound healing and the treatment of wrinkles (Sekhon-Randhawa and Rahman 2014).

Genetic factors have been shown to be a key target for the improvement of rhamnolipid production by *P. aeruginosa* (Dobler et al. 2016; Gutiérrez-Gómez et al. 2018; Huang et al. 2018). However, rhamnolipid production is highly affected by medium composition and cultivation conditions. Several studies had focused on exploring various fermentation strategies for enhancing rhamnolipid yields by optimizing fermentation parameters. For instance, compared to water-soluble carbon sources (e.g., glucose), the use of water-insoluble carbon such as vegetable oil generally produces rhamnolipids in higher titers (Chong and Li 2017). The use of factorial design to optimize culture conditions has been proven to be a successful approach to significantly increase the rhamnolipid yield obtained using several *P. aeruginosa* strains (Camilios-Neto et al. 2008; Kumar et al. 2015; Ozdal et al. 2017; El-Housseiny et al. 2019). Various studies (see Table 1) have explored economical production methods using low-cost and readily available nutrients. Carbon and nitrogen sources are among the most important parameters for rhamnolipid production, with an appropriate C/N ratio being a key factor (Huang et al. 2018). To develop a cost-effective bioprocess, cheap nutrient sources are needed and complex mineral medium cannot be used. The use of domestic or industrial wastes for rhamnolipid production is both cost-effective and environmental-friendly (Ozdal et al. 2017).

**Table 1:** Rhamnolipid production using *P. aeruginosa* as producer microorganism with different waste and non-waste carbon sources, nitrogen sources, culture media and production time

Highest rhamnolipid yield (g/L)	*P. aeruginosa* strain	Culture medium	Carbon source	Nitrogen source	Time	References
2.70	47T2 NCIB 40044	NaNO_3_, KH_2_PO_4_, K_2_HPO_4_, KCl, MgSO_4_·7H_2_O, CaCl_2_, FeSO_4_.7H_2_O, yeast extract and trace elements	40 g/L waste frying vegetable oils	4 g/L NaNO_3_	80 h	Haba et al. (2000)
1.82	PEER02	KCl, NaCl, FeSO_4_∙7H_2_O, KH_2_PO_4_, K_2_HPO_4_, MgSO_4_∙7H_2_O, yeast extract, trace elements	2% v/v soy oil	15 g/L NaNO_3_	4 days	Wang et al. (2007)
9.50	MR01	KH_2_PO_4_, MgSO_4_·7H_2_O, yeast extract.	4% v/v soy oil	0.2% w/v NaNO_3_	336/360 h	Lotfabad et al. (2010)
3.55	D	KH_2_PO_4_, Na_2_HPO_4_, MgSO_4_·7H_2_O, glycerol, yeast extract.	2% waste coconut oil	6.5 g/L NaNO_3_	7 days	George and Jayachandran. (2012)
0.89	PA01	Glucose, Na_2_HPO_4_, KH_2_PO_4_, 0.4 MgSO_4_·7H_2_O, CaCl_2_·2H_2_O, FeSO_4_·7H_2_O, and trace elements	2% w/v waste oil	2 g/L NaNO_3_	7 days	Moya-Ramírez et al. (2015)
4.53	2297	KH_2_PO_4_, K_2_HPO_4_, MgSO_4_·7H_2_O	2% sawdust	1 g/L (NH_4_)_2_SO_4_	120 h	Kumar et al. (2015)
2.16	Local isolate (wild-type)	Not specified	1% v/v glycerol	2% w/v NaNO_3_	54 h	Eraqi et al. (2016)
4.5–5.1	Wild-type strain	Oil mill wastewater (25% v/v)	Corn steep liquor (10% w/v); Sugar cane molasses (10% w/v)	Not specified		Gudiña et al. (2016)
2.80	DR1	MgSO_4_·7H_2_O, NaCl, KCl, CaCl_2_·2H_2_O, H_3_PO_4_, trace elements	1% mango kernel oil, 1% glucose	2.5 g/L NaNO_3_	96 h	Sathi-Reddy et al. (2016)
5.00	L05	Na_2_HPO_4_, KH_2_PO_4_, K_2_HPO_4_, trace elements	19.43 mM of myristic acid	1.4 g/L NaNO_3_	144 h	Nicolo et al. (2017)
5.53	AMB	Na_2_HPO_4_, KH_2_PO_4_, NaCl, MgSO_4_·7H_2_O, CaCl_2_·2H_2_O	2% w/v waste coconut oil	0.1 g/L NaNO_3_	60 h	Samykannu and Achary (2017)
41.87	15GR	MgSO_4_·7H2O, NaCl, KCl, CaCl_2_·2H_2_O, H_3_PO_4_, FeSO_4_·7H2O, ZnSO_4_·7H_2_O, MnSO_4_·H_2_O, K_3_BO_3_, CuSO_4_·5H_2_O, Na_2_MoO_4_·2H_2_O	2% v/v glycerol	2.5 g/L NaNO_3_	6 days	El-Housseiny et al. (2019)

The highest rhamnolipid yield and the used *P. aeruginosa* strain in each case is reported

Large amounts of waste cooking oil are produced each year in homes and restaurants and by the food industry (Chhetri et al. 2008). The improper disposal of waste cooking oil can cause serious environmental contamination as well as operational problems in sewers and water treatment plants (Panadare and Rathod 2015). However, the adequate management of waste cooking oil and its use as a feedstock to generate industrial subproducts can have economic and environmental benefits (Patil et al. 2012). Recycled waste cooking oil is primarily used as feedstock for biodiesel generation, although it has numerous other applications (Panadare and Rathod 2015). Relatively few studies have investigated the production of rhamnolipids by *P. aeruginosa* using waste cooking oils as a carbon source (see Table 1; Chong and Li 2017), and to the best of our knowledge, waste canola oil has yet to be investigated for this purpose.

The goal of this study was to evaluate the potential of waste canola oil as a cost-effective and environmentally friendly carbon source for rhamnolipid production by *P. aeruginosa* in combination with an adequate nitrogen source through a full factorial experimental design.

## Materials and methods

### Molecular identification of the bacterial strain

The *P. aeruginosa* strain used in this study was donated by a Mexican Medical laboratory (http://laboratoriosruiz.com/), where it was previously identified as *P. aeruginosa* using the Vitek 2 GN typing panel (Biomérieux, México) and analyzed using the Vitek 2 instrument (Biomérieux, México). The identity of the strain was confirmed by sequencing a portion of the 16S rRNA gene with bacterial-specific primers Bacfw (ACTCCTACGGGAGGCAG) and Bacrev (GACTACCAGGGTATCTAATCC) (Yu et al. 2005). The strain was cultivated in 50 mL of nutritive broth (BIOXON, Mexico) for 13 h at 37 °C with shaking at 200 rpm. DNA extraction was performed using a QIAamp DNA Mini kit (Qiagen, Mexico) according to the manufacturer instructions. PCR amplification was performed in a reaction containing 12.5 µL PrimeSTAR polymerase mix, 1 µL forward primer (10 µM), 1 µL reverse primer (10 µM), 4 µL genomic DNA as template (5 ng), and 6.5 µL MiliQ water. The thermocycling program was as follows: (1) 98 °C for 5 min, (2) 98 °C for 10 s, (3) 58 °C for 15 s, (4) 72 °C for 25 s, (5) repeat steps 2, 3 and 4 30× and a final extension at 72 °C for 5 min. The PCR product was purified using a QIAquick PCR Purification kit (Qiagen, Mexico) according to the manufacturer’s instructions. The purified PCR product was sequenced in triplicate using the Sanger technique at the Biotechnology Institute of the National Autonomous University of Mexico). The consensus sequence was deduced from the obtained sequences using BioEdit, and the identity of the sequence was determined using the program BLAST.

### Inoculum conditions

A 500-ml shake flask containing 100 mL of nutritive broth (BIOXON, Mexico) was inoculated with a 5% (v/v) overnight culture of *P. aeruginosa* with a starting OD_565nm_ of 0.012 (nutritive broth). The culture was incubated at 37 °C with shaking at 200 rpm in a Benchmark Incu-Shaker until the optical density at 565 nm reached 0.745 ± 0.020 (corresponding to 1.53 × 10^8^ CFU/mL), indicating that the culture was in mid-exponential phase according to the logistic growth model (Additional file 1: Figure S1). The culture was used as an inoculum for rhamnolipid production.

### Factorial design

Rhamnolipid production was optimized using four different 2^3^ full factorial design with three independent factors, including carbon source, nitrogen source and production time. Factorial design 1 used canola oil as a carbon source at two concentrations (1 and 3%, v/v), (NH_4_)_2_SO_4_ as a nitrogen source at two concentrations (1 and 4 g/L), and production times of 7 and 14 days. These 2 incubation times were selected based on previously published research where production times varied between 7 (George and Jayachandran. 2012; Moya-Ramírez et al. 2015) and 14 days (Lotfabad et al. 2010). Factorial design 2 used waste canola oil as a carbon source at two concentrations (1 and 3%, v/v), (NH_4_)_2_SO_4_ as a nitrogen source at two concentrations (1 and 4 g/L), and production times of 7 and 14 days. Factorial design 3 used canola oil as a carbon source at two concentrations (1 and 3%, v/v), NaNO_3_ as a nitrogen source at two concentrations (1 and 4 g/L), and production times of 7 and 14 days. Factorial design 4 used waste canola oil as a carbon source at two concentrations (1 and 3%, v/v), NaNO_3_ as a nitrogen source at two concentrations (1 and 4 g/L), and production times of 7 and 14 days. Eight experiments were performed for each factorial design in two replicates. The measured response variable was rhamnolipid production, which was expressed as rhamnose equivalents (mg/L). Sixty-four experiments in total were performed for the four different 2^3^ experimental designs. Minitab statistical software was used to statistically analyze the data (*p *< 0.05). Waste canola oil was obtained from a University restaurant and was filtered prior to its use to remove any particles. The experimental design is summarized in Table 2.

**Table 2:** 2^3^ full factorial designs used to optimize rhamnolipid production

Factorial design 1			
Treatment	Canola oil (% v/v)	(NH_4_)_2_SO_4_ (g/L)	Production time (days)
T1	1	1	7
T2	3	1	7
T3	1	4	7
T4	3	4	7
T5	1	1	14
T6	3	1	14
T7	1	4	14
T8	3	4	14

Factorial design 2			
Treatment	Waste canola oil (% v/v)	(NH_4_)_2_SO_4_ (g/L)	Production time (days)
T1	1	1	7
T2	3	1	7
T3	1	4	7
T4	3	4	7
T5	1	1	14
T6	3	1	14
T7	1	4	14
T8	3	4	14

Factorial design 3			
Treatment	Canola oil (% v/v)	NaNO_3_ (g/L)	Production time (days)
T1	1	1	7
T2	3	1	7
T3	1	4	7
T4	3	4	7
T5	1	1	14
T6	3	1	14
T7	1	4	14
T8	3	4	14

Factorial design 4			
Treatment	Waste canola oil (% v/v)	NaNO_3_ (g/L)	Production time (days)
T1	1	1	7
T2	3	1	7
T3	1	4	7
T4	3	4	7
T5	1	1	14
T6	3	1	14
T7	1	4	14
T8	3	4	14

### Culture conditions

The experiments were carried out in Erlenmeyer flasks (500 mL) containing 100 mL of an autoclave sterilized mineral medium containing 1 g/L K_2_HPO_4_, 1 g/L KH_2_PO_4_, and 0.41 g/L MgSO_4_·7H_2_O supplemented with the corresponding carbon and nitrogen sources according to the factorial design. The culture medium was inoculated with a 5% (v/v) of mid-exponential growth culture (OD_565nm_ = 0.745 ± 0.020; 1.53 × 10^8^ CFU/mL). The cultures were incubated at 37 °C with shaking at 200 rpm in a Benchmark Incu-Shaker.

### Rhamnolipid extraction

Rhamnolipid extraction was performed using the method described by Camilios-Neto et al. (2008) with modifications. At the end of the experiments, the culture broth was centrifuged at 5000 rpm for 20 min (Hermle Labnet Z 326), and the supernatant was recovered and mixed with one volume of chloroform/ethanol (3:1 v/v). After mixing, phase separation was performed in a separation funnel. The lower organic phase was collected, and the process was repeated with the upper aqueous phase until no emulsification was observed. The organic phase was evaporated in a rotary evaporator (HAHNVAPOR, HS-2001NS) at 40 °C, and the rhamnolipids were resuspended in one volume of distilled water.

### Rhamnolipid quantification

**Fig. 1:**
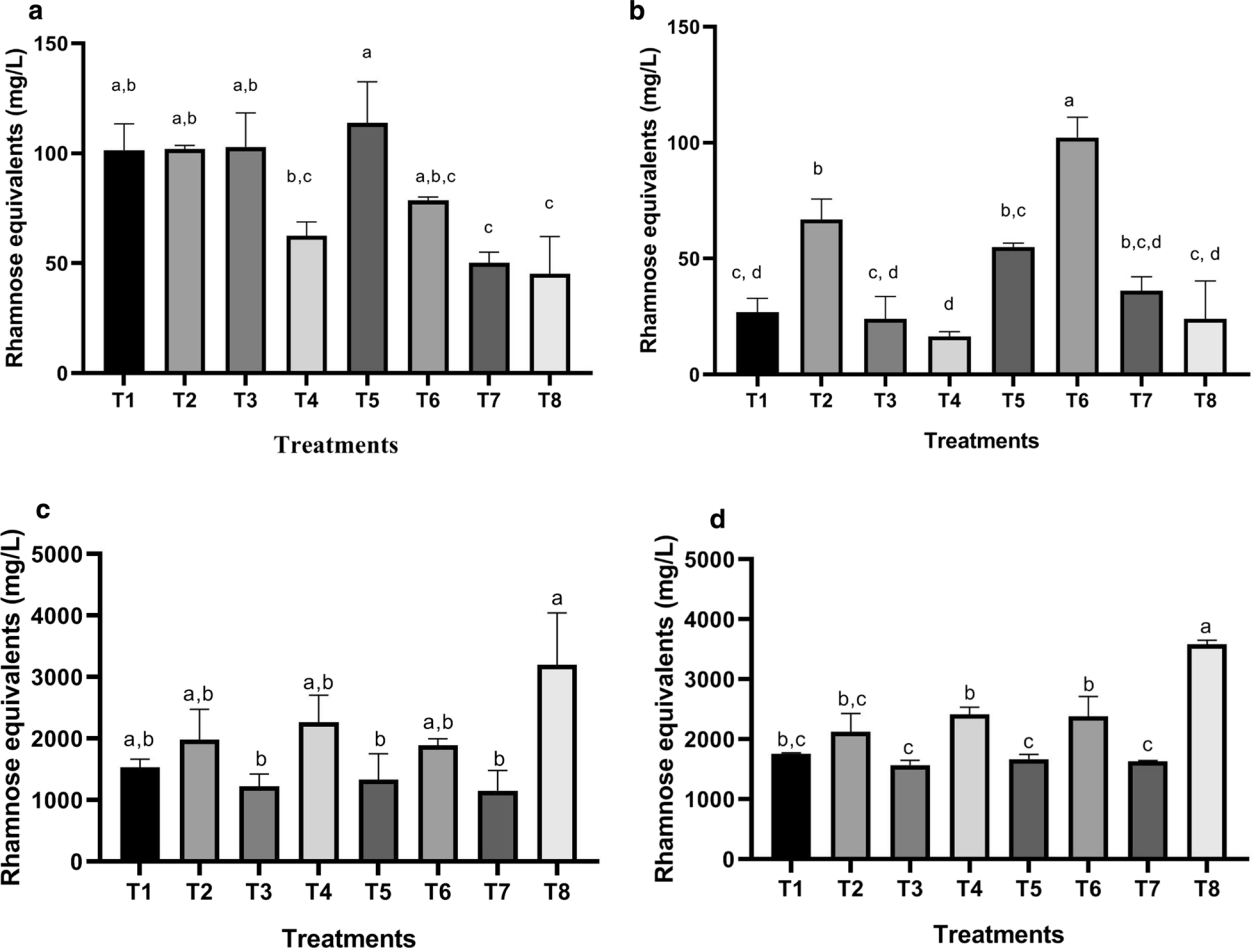
The four full factorial designs used to optimize rhamnolipid production. The rhamnolipid yield is expressed as rhamnose equivalents. **a** Factorial design 1 (Canola Oil/(NH_4_)_2_SO_4_); **b** Factorial design 2 (Waste canola Oil/(NH_4_)_2_SO_4_); **c** Factorial design 3 (Canola Oil/(NaNO_3_); **d** Factorial design 4 (Waste canola Oil/(NaNO_3_). Treatments that do not share a letter are significantly different (*p *< 0.05)

The rhamnolipids were quantified as described by Wang et al. (2007). Briefly, a 9:1 (v/v) mixture of orcinol (0.19% in 53% H_2_SO_4_) and rhamnolipids sample was warmed in a water bath (Terlab, TE-B80D) at 80 °C for 30 min. Subsequently, the sample was cooled for 10 min in a water bath at room temperature. The optical density was measured at 421 nm using a UV–Vis spectrophotometer. The rhamnolipid concentration was obtained using a standard curve of l-rhamnose (0–50 mg/L), and the results were expressed as rhamnose equivalents (mg/L). The data corresponding to Fig. 1 can be found in Additional file 1: Figure S2.

## Results

### Confirmation of the identity of the bacterial strain

The bacterial strain used in this study was previously identified as *P. aeruginosa* using automated biochemical tests. To confirm the identity of the strain, a partial 16S rRNA gene sequence was obtained (NCBI accession number MK307837). The sequence was analyzed using Megablast and exhibited 100% similarity with the 16S rRNA gene sequences of several *P. aeruginosa* strains.

### Full factorial design results

Four full factorial designs were used to evaluate the performance of waste canola oil compared with canola oil as well as to determine the optimal nitrogen source, carbon/nitrogen ratio and production time (Fig. 1).

When canola oil and ammonium sulfate were used as carbon and nitrogen sources, respectively (Factorial design 1; Fig. 1a), no significant differences in rhamnolipid yields (*p* < 0.05) were observed between treatments 1, 2, 3, 5 and 6. The highest rhamnolipid yield was observed for treatment 5 (113.83 ± 18.69 mg/L). Upon switching the carbon source to waste canola oil, while the nitrogen source remained ammonium sulfate (Factorial design 2; Fig. 1b), treatment 6 (High carbon, low nitrogen, 14 days) showed the highest rhamnolipid yield (102.24 ± 8.66 mg/L).

When canola oil and sodium nitrate were used as carbon and nitrogen sources, respectively (Factorial design 3; Fig. 1c), the highest rhamnolipid yield (3196.33 ± 848.05 mg/L) was obtained for treatment 8 (high carbon and nitrogen, 14 days) and was significantly different from the other treatments. Upon switching the carbon source to waste canola oil, while the nitrogen source remained as sodium nitrate (Factorial design 4; Fig. 1d), the highest rhamnolipid yield (3585.31 ± 66.24 mg/L) was also observed with treatment 8 and was significantly different from the other treatments. The highest yield among the 32 treatments of the 4 factorial designs was obtained for treatment 8 of factorial design 4.

The use of sodium nitrate as a nitrogen source (factorial designs 3 and 4) led to a 30-fold increase in rhamnolipid yield under all treatments (Fig. 1c, d) compared to the yield obtained when ammonium nitrate was used as a nitrogen source (factorial designs 1 and 2; Fig. 1a, b). The rhamnolipid yield obtained when waste canola oil was used as a carbon source (Fig. 1d) was comparable and even slightly higher than the yield observed when canola oil was used as a carbon source (Fig. 1c).

## Discussion

The *P. aeruginosa* strain used in this study was a wild-type clinical isolate that was previously identified using biochemical tests. Partial sequencing of the 16S rRNA gene in combination with the biochemical tests results enabled the confirmation of the identity of the strain as *P. aeruginosa.* This species is considered a metabolically versatile bacterium that is able to use a variety of simple and complex carbon sources and can survive under normal and harsh environmental conditions (Rojo 2010; El-Housseiny et al. 2019). These characteristics make *P. aeruginosa* an excellent candidate for rhamnolipid production using unusual carbon sources.

The results of numerous reports indicate that rhamnolipid production by *P. aeruginosa* is dependent on medium components in addition to other factors (Mulligan and Gibbs 1989; Müller et al. 2012; El-Housseiny et al. 2016, Gutiérrez-Gómez et al. 2018), with carbon and nitrogen sources being among the most important factors to be considered (Huang et al. 2018). To determine the potential of waste canola oil as a carbon source for rhamnolipid production, we used a factorial design to determine the effect of 3 parameters, carbon source (canola oil and waste canola oil), nitrogen source (ammonium sulfate and sodium nitrate) and production time (7 and 14 days) as well as the interaction between these parameters (Table 2). The results presented in Fig. 1 show that when ammonium nitrate was used as a nitrogen source, rhamnolipid production remained low regardless of the carbon source and production time used, with canola oil faring slightly better than waste canola oil (Fig. 1a, b). However, when the nitrogen source was switched to sodium nitrate a 12 to 30-fold increase in rhamnolipid production was observed (Fig. 1c, d). This result is in agreement with previously published reports. Most studies favor the use of nitrate over ammonium as a nitrogen source for rhamnolipid production by *P. aeruginosa* (see Table 1). Furthermore, it was shown that under certain experimental conditions, sodium nitrate promotes rhamnolipid production, whereas ammonium sulfate inhibits it (Mulligan and Gibbs 1989). However, alternative nitrogen sources should not be entirely discounted, as they may still be useful under certain circumstances (Huang et al. 2018), including ammonium sulfate (Kumar et al. 2015). Thus, the best course of action is to determine the nitrogen source that pairs best with the desired carbon source to maximize rhamnolipid yield.

When sodium nitrate was used as a nitrogen source, waste canola oil (Fig. 1d) performed as well and even slightly better than canola oil (Fig. 1c), particularly under experimental conditions using high concentrations of carbon and nitrogen, and 14 days of production (treatment T8, Fig. 1c, d). The maximum rhamnolipid yield attained under these conditions (3.6 g/L for waste canola oil and 3.2 g/L for canola oil) is comparable to that obtained by other studies using a variety of different carbon sources (see Table 1), particularly when the use of submerged fermentation conditions and wild-type strains are considered. These results indicates that waste canola oil has excellent potential as a carbon source for the production of rhamnolipids by *P. aeruginosa.* Importantly, only three parameters were considered in our study for the optimization of culture conditions (carbon source, nitrogen source and production time). Thus, additional improvements could be made in the rhamnolipid yield obtained using waste canola oil as a carbon source through the optimization of other important bioprocess parameters, such as pH, incubation temperature and dissolved oxygen among others (Zhu et al. 2012; Müller et al. 2012; Bazsefidpar et al. 2019).

The fact that the highest rhamnolipid yield was obtained for treatment 8 for factorial designs 3 (Fig. 1c) and 4 (Fig. 1d) suggests that the C/N ratio under these experimental conditions is the closest to the optimal C/N ratio when canola or waste canola oil is used as a carbon source and sodium nitrate is used as a nitrogen source. Interestingly, when rhamnolipid production time was set to 7 days (treatments 1, 2, 3 and 4), the highest yield was obtained for treatment 4 that have the same C/N ratio as treatment 8, regardless of the used carbon source (Fig. 1c, d). Both treatments 4 and 8 feature the highest carbon and nitrogen contents but differ by the production times: 7 and 14 days respectively (Table 1). The fact that the rhamnolipid yield was higher for treatment 8 as compared to treatment 4 suggests that both carbon and nitrogen sources where limiting for all other treatments. These results suggest that further improvements of rhamnolipid yield could be made through the optimization of the C/N ratio and the used carbon and nitrogen substrates concentration.

When vegetable oil is fried, it undergoes several types of chemical degradation reactions that result in a change in its fatty acid composition and physicochemical characteristics, including hydrolysis, oxidation and polymerization (Choe and Min 2007). The first two reactions lead to an increase in the free fatty acid content of the oil and a decrease in the degree of unsaturation of fatty acids, respectively. These reactions are correlated with the greater acid value and higher viscosity observed with used cooking oil compared with unused vegetable oil (Chhetri et al. 2008; Knothe and Steidley 2009). Knothe and Steidley (2009) observed an overall increase in the saturation of fatty acids in used vegetable oils compared to the corresponding oil before use, with an observed increase in stearic (C18:0) and oleic (C18:1) acids and a decrease in linoleic (C18:2) and linolenic (C18:3) acids. Huang et al. 2018 tested various vegetable oils as carbon sources for rhamnolipid production, including palm, olive, rapeseed (canola), soybean and corn oils. Their results showed that olive oil was the carbon source that produced the highest rhamnolipid yield under their experimental conditions. Since oleic acid is by far the most abundant fatty acid in olive oil (Gunstone 1996), their results might indicate a preference of *P. aeruginosa* for oleic acid. Canola oil has a typical fatty acid composition of approximately 60% oleic acid, 20% linoleic acid and 10% linolenic acid (Warner and Mounts 1993). In contrast, olive oil generally has a higher content of oleic acid (78%) and a lower content of linoleic (7%) and linolenic (1%) acids than canola oil (Gunstone 1996). When canola oil is fried, an increase in oleic acid and a decrease in linoleic and linolenic acid contents would confer a fatty acid profile to the waste canola oil that is globally similar to that of unused olive oil. This phenomenon may explain the increase in rhamnolipid production when waste canola oil is used as a carbon source (Fig. 1d) compared to the yield obtained with unused canola oil (Fig. 1c). The increase in free fatty acid content in waste cooking oil would be challenging for any bacterial species due to its antibacterial effects (Yoon et al. 2018). However, *P. aeruginosa* is resistant to most free fatty acids other than eicosapentaenoic acid (C20:5), making *P. aeruginosa* particularly suitable for rhamnolipid production using waste cooking oil.

It is particularly noteworthy that the rhamnolipid yield attained in this study was achieved using a mineral medium that contained only four salts (K_2_HPO_4_, KH_2_PO_4_, MgSO_4_ and NaNO_3_) included waste canola oil as the sole carbon source. This medium is considered minimal when compared to the composition of the more complex growth media used in other studies (see Table 1). Along with cost reductions due to the use of waste canola oil as a carbon source, the use of a simplified mineral medium should lead to further cost reductions upon scaling-up the bioprocess. High production costs are one of the major obstacles facing the large-scale industrial production of biosurfactants (Müller et al. 2012; Chong and Li 2017). Thus, increasing the rhamnolipid yield should be pursued in parallel with the development of low-cost substrates and growth media to increase the economic and technical feasibility of the industrial production of biosurfactants.

## Additional files

**Additional file 1.** Additional figures.

**Additional file 2.** Additional tables.
