# Supplementary material for: Use of waste canola oil as a low-cost substrate for rhamnolipid production using Pseudomonas aeruginosa
Source: AMB Express. 2019 May 6;9:61. doi: 10.1186/s13568-019-0784-7 (PMC6502917; doi:10.1186/s13568-019-0784-7)
Supplement: Supplementary file 2 — Additional file 2. Additional tables. [file 13568_2019_784_MOESM2_ESM.docx]

**Data corresponding to the results of the four experimental designs presented in Figure 1.**

| **Factorial design 1** | | | |
| --- | --- | --- | --- |
| Treatment | canola oil, (NH_4_)_2_SO_4_, and time | Rhamnolipid yield (rhamnose equivalent, mg/L) | Tukey Pairwise Comparisons  (p <0.05) |
| 1 | 1% 1g/L 7d | 101.36 ± 12.03 | A, B |
| 2 | 3% 1g/L 7d | 101.90 ± 1.73 | A, B |
| 3 | 1% 4g/L 7d | 102.82 ± 15.63 | A, B |
| 4 | 3% 4g/L 7d | 62.59 ± 6.25 | B, C |
| **5** | **1% 1g/L 14d** | **113.83 ± 18.69** | **A** |
| 6 | 3% 1g/L 14d | 78.62 ± 1.52 | A, B, C |
| 7 | 1% 4g/L 14d | 50.17 ± 4.86 | C |
| 8 | 3% 4g/L 14d | 45.31 ± 16.84 | C |
| **Factorial design 2** | | | |
| Treatment | Waste canola oil, (NH_4_)_2_SO_4_, and time | Rhamnolipid yield (rhamnose equivalent, mg/L) | Tukey Pairwise Comparisons  (p <0.05) |
| 1 | 1% 1g/L 7d | 26.97 ± 5.92 | C, D |
| 2 | 3% 1g/L 7d | 66.87 ± 8.85 | B |
| 3 | 1% 4g/L 7d | 24.05 ± 9.57 | C, D |
| 4 | 3% 4g/L 7d | 16.53 ± 2.02 | D |
| 5 | 1% 1g/L 14d | 54.90 ± 1.73 | B, C |
| **6** | **3% 1g/L 14d** | **102.24 ± 8.66** | **A** |
| 7 | 1% 4g/L 14d | 36.26 ± 5.96 | B, C, D |
| 8 | 3% 4g/L 14d | 24.06 ± 16.33 | C, D |
| **Factorial design 3** | | | |
| Treatment | Canola oil, NaNO_3_, and time | Rhamnolipid yield (rhamnose equivalent, mg/L) | Tukey Pairwise Comparisons  (p <0.05) |
| 1 | 1% 1g/L 7d | 1529.83 ± 136.08 | A, B |
| 2 | 3% 1g/L 7d | 1987.04 ± 491.37 | A, B |
| 3 | 1% 4g/L 7d | 1222.33 ± 202.15 | B |
| 4 | 3% 4g/L 7d | 2266.07 ± 438.33 | A, B |
| 5 | 1% 1g/L 14d | 1330.87 ± 422.05 | B |
| 6 | 3% 1g/L 14d | 1888.23 ± 108.47 | A, B |
| 7 | 1% 4g/L 14d | 1150.98 ± 330.67 | B |
| **8** | **3% 4g/L 14d** | **3196.33 ± 848.05** | **A** |
| **Factorial design 4** | | | |
| Treatment | Waste canola oil, NaNO_3_, and time | Rhamnolipid yield (rhamnose equivalent, mg/L) | Tukey Pairwise Comparisons  (p <0.05) |
| 1 | 1% 1g/L 7d | 1758.54 ± 13.15 | B, C |
| 2 | 3% 1g/L 7d | 2125.10 ± 307.81 | B, C |
| 3 | 1% 4g/L 7d | 1570.27 ± 78.23 | C |
| 4 | 3% 4g/L 7d | 2414.39 ± 122.08 | B |
| 5 | 1% 1g/L 14d | 1667.43 ± 80.20 | C |
| 6 | 3% 1g/L 14d | 2386.84 ± 327.29 | B |
| 7 | 1% 4g/L 14d | 1631.17 ± 11.83 | C |
| **8** | **3% 4g/L 14d** | **3585.31 ± 66.24** | **A** |
